# Association of diabetes mellitus and glycemic control with left ventricular function and deformation in patients after acute myocardial infarction: a 3 T cardiac magnetic resonance study

**DOI:** 10.1186/s12933-023-01785-9

**Published:** 2023-03-11

**Authors:** Yue Gao, Rui Shi, Yuan Li, Ying-kun Guo, Hua-Yan Xu, Ke Shi, Zhi-gang Yang

**Affiliations:** 1 Department of Radiology, West China Hospital, Sichuan University, 37# Guo Xue Xiang, Chengdu, 610041 Sichuan China; 2 Department of Radiology, Key Laboratory of Birth Defects and Related Diseases of Women and Children of Ministry of Education, West China Second University Hospital, Sichuan University, Chengdu, Sichuan China

**Keywords:** Diabetes mellitus, Myocardial infarction, Global peak strain, Cardiovascular magnetic resonance

## Abstract

**Background:**

Diabetes mellitus (DM) is considered a major risk factor for myocardial infarction (MI), and MI patients with DM have a poor prognosis. Accordingly, we aimed to investigate the additive effects of DM on LV deformation in patients after acute MI.

**Materials and methods:**

One hundred thirteen MI patients without DM [MI (DM−)], 95 with DM [MI (DM+)] and 71 control subjects who underwent CMRscanning were included. LV function, infarct size and LV global peak strains in the radial, circumferential and longitudinal directions were measured. MI (DM+) patients were divided into two subgroups based on the HbA1c level (< 7.0% and ≥ 7.0%). The determinants of reduced LV global myocardial strain for all MI patients and MI (DM+) patients were assessed using multivariable linear regression analyses.

**Results:**

Compared with control subjects, both MI (DM−) and MI (DM+) patients presented higher LV end-diastolic and end-systolic volume index and lower LV ejection fraction. LV global peak strains progressively declined from the control group to the MI(DM−) group to the MI(DM+) group (all *p* < 0.05). Subgroup analysis showed that LV global radial PS and longitudinal PS were worse in MI(MD+) patients with poor glycemic control than in those with good glycemic control (all *p* < 0.05). DM was an independent determinant of impaired LV global peak strain in radial, circumferential and longitudinal directions in patients after acute MI (β = − 0.166, 0.164 and 0.262, both *p* < 0.05). The HbA1c level was independently associated with a decreased LV global radial PS (β =  − 0.209, *p* = 0.025) and longitudinal PS (β = 0.221, *p* = 0.010) in MI (DM+) patients.

**Conclusions:**

DM has an additive deleterious effect on LV function and deformation in patients after acute MI, and HbA1c was independently associated with impaired LV myocardial strain.

## Introduction

Coronary artery disease and myocardial infarction (MI) are major causes of global morbidity and mortality [1]. Assessment and management of risk factors are the core of the treatment strategy for MI patients. Diabetes mellitus (DM) is considered a major risk factor for coronary artery disease, and patients with DM are at a high risk of MI and have a poor prognosis [2–4]. Left ventricular (LV) hypertrophy, myocardial fibrosis, and diastolic and systolic dysfunction are the main causes of diabetic cardiomyopathy [5–7]. Diastolic dysfunction is one of the important indicators of early left ventricular (LV) dysfunction before reduced LV ejection fraction in DM patients, which can earlier indicate the possible occurrence of ischemic events [8, 9].

Previous studies have noted that both MI and DM can lead to LV dysfunction and impaired deformation, culminating in the progressive deterioration of HF and poor outcomes [10, 11]. Meanwhile, hyperglycemia status can aggravate cardiac structural and functional abnormalities, such as replacement myocardial fibrosis and LV wall stiffness [12, 13]. Therefore, among patients after acute MI, investigating the effects of DM and glycemic control on LV myocardial deformation is important to achieve the goal of health management.

Cardiac magnetic resonance (CMR) imaging provides comprehensive information on cardiac function, deformation, and myocardial tissue. Deformation, especially impaired global longitudinal strain, has been proven to be associated with cardiovascular events and has better prognostic value than LVEF [14, 15]. Therefore, the current study sought to investigate the additive effects of DM on LV function and global deformation in patients after acute MI.

## Methods and materials

### Study population

The study protocol was approved by the Biomedical Research Ethics Committee of our hospital. Informed consent was waived due to the retrospective nature of the research.

**Fig. 1:**
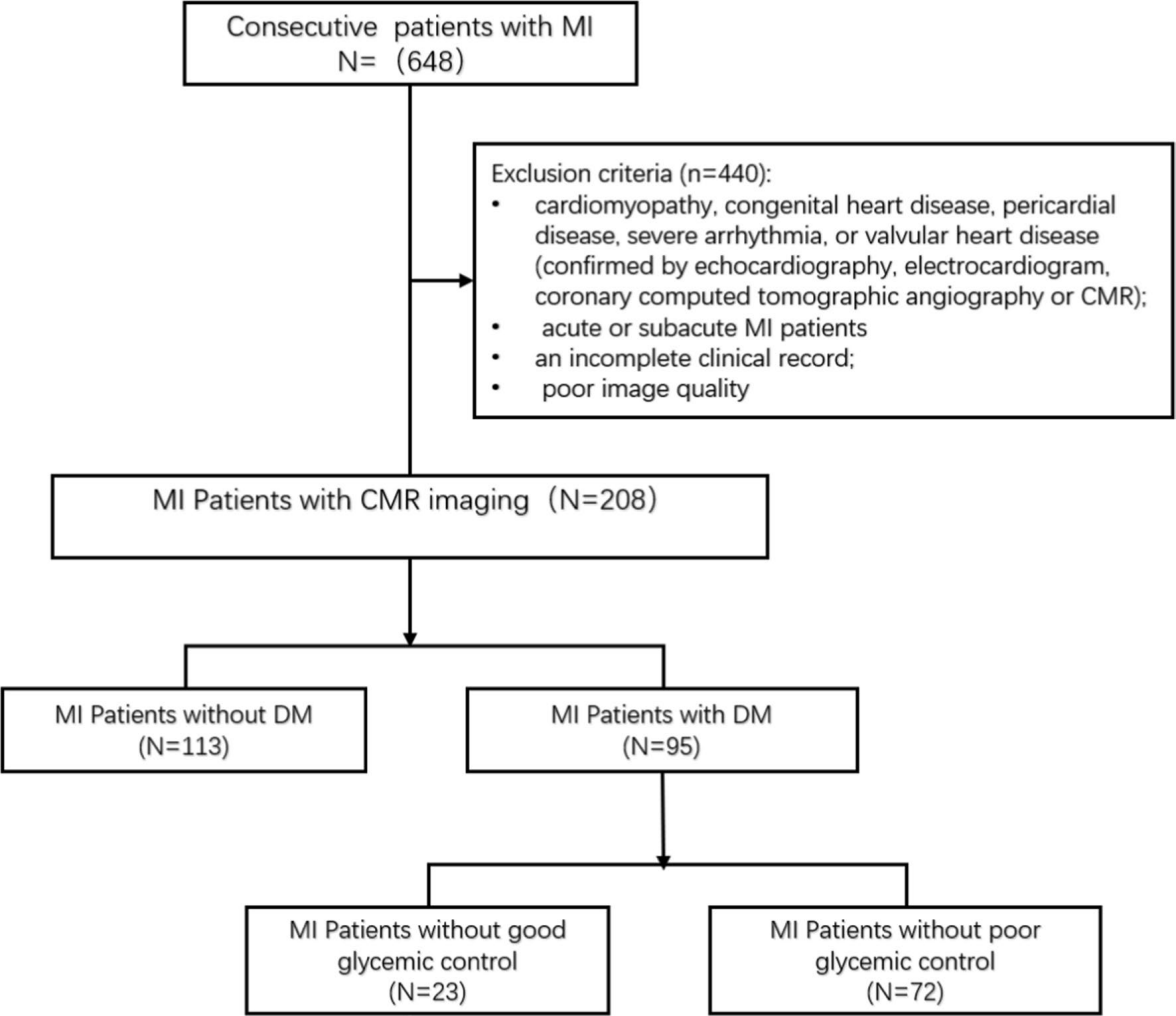
Flow chart of the study

Initially, we consecutively retrospectively enrolled 648 patients with MI who had completed CMR examinations in our hospital between January 2010 and March 2022. MI was diagnosed in our hospital and meet the diagnostic criteria for the universal definition of MI (2007, 2012 and 2018, which based on clinical symptoms, electrocardiogram changes, and creatine kinase and/or troponin T levels greater than standards), and have a history of MI by clinically diagnosed. The exclusion criteria were as follows: (1) cardiomyopathy, congenital heart disease, pericardial disease, severe arrhythmia, or valvular heart disease (confirmed by echocardiography, electrocardiogram, coronary computed tomographic angiography or CMR); (2) acute or subacute MI patients [16]; (3) an incomplete clinical record; and (4) inadequate images because of arrhythmia or poor image quality. Following these criteria, a total of 208 patients after MI were included in this study. According to whether there was coexisting DM, patients were further divided into MI (DM+) and MI (DM−) groups. The diagnosis of DM was based on current European Society of Cardiology (2019) guidelines [17]. The treatment, culprit vessel and diseased coronary artery of all MI patients were recorded. To evaluate the influence of glycemic control on LV, MI(DM+) patients were categorized as having good glycemic control (HbA1c < 7.0%) or poor glycemic control (HbA1c ≥ 7.0%). A detailed flow chart of the present study is presented in Fig. 1. In addition, age-, sex-, and body mass index-matched subjects without a history of MI were enrolled as controls. Exclusion criteria for the control group were as follows: (1) DM or impaired glucose tolerance; (2) presence of dyspnea, chest pain, palpitation, or other cardiovascular disease-related symptoms; (3) electrocardiogram abnormalities; and (4) CMR detected abnormalities (perfusion defect, local or diffuse myocardial late gadolinium enhancement, abnormal ventricular motion, valvular stenosis, etc.). Finally, a total of 71 controls were included in this study.

### CMR scanning protocol

All CMR examinations were performed in the supine position using a 3.0 T whole body magnetic resonance scanner Trio Tim or MAGNETOM Skyra (Siemens Medical Solutions, Erlangen, Germany) equipped with 32-channel body phased array coils and standard ECG trigger equipment. Balanced steady-state free precession (b-SSFP) cine images were acquired using a retrospective vector ECG gating technique at the end of inspiratory breath holding, and twenty-five frames were reconstructed per breath-hold acquisition. Standard short-axis, long-axis two- and four-chamber cine images were obtained. that covered the entire left ventricles. The following scanning parameters were used: repetition time (TR) 2.8 ms or 3.4 ms, echo time (TE) 1.22 ms, flip angle 40° or 50°, slice thickness 8 mm, field of view (FOV) 250 × 300 mm^2^ or 340 × 285mm^2^, and matrix 208 × 139 or 256 × 166. Gadolinium-based contrast agent was intravenously injected at a dose of 0.2 mmol/kg body weight at an injection rate of 2.5–3.0 mL/s, followed by a 20 mL saline flush at a rate of 3.0 mL/s. LGE images were acquired in the corresponding slice position as the cine imaging 10–15 min after contrast injection. The images were obtained using a phase-sensitive inversion recovery sequence with the following parameters: TR 750/512 ms, TE 1.18/1.24 ms, flip angle 40°, slice thickness 8 mm, FOV 240 × 300 mm^2^ or 288 × 360 mm^2^, and matrix 256 × 184 mm^2^ or 256 × 125 mm^2^.

### CMR data analysis

All CMR data were uploaded to an offline workstation using semiautomated software (Cvi42; Circle Cardiovascular Imaging, Inc., Calgary, Canada). The LV endocardial and epicardial traces were manually or semiautomatically delineated in serial short-axis slices at the end-diastolic and end-systolic phases. Papillary muscles were considered part of the ventricular cavity and LV mass, and epicardial fat was excluded. LV functional parameters, including LV end-diastolic volume (LVEDV), LV end-systolic volume (LVESV), LV stroke volume (LVSV), LVEF and LV mass (LVM), were computed automatically. LVEDV, LVESV, LVSV and LVM were indexed to body surface area (BSA). The LV global function index (LVGFI) was calculated using the following formula:

$${\text{LVGFI}} = \left\{ {{\text{LVSV/ }}\left[ {\left( {{\text{LVEDV}} + {\text{LVESV}}} \right)/2 + \left( {{\text{LVM}}/1.05} \right)} \right]} \right\} \times 100$$

**Fig. 2:**
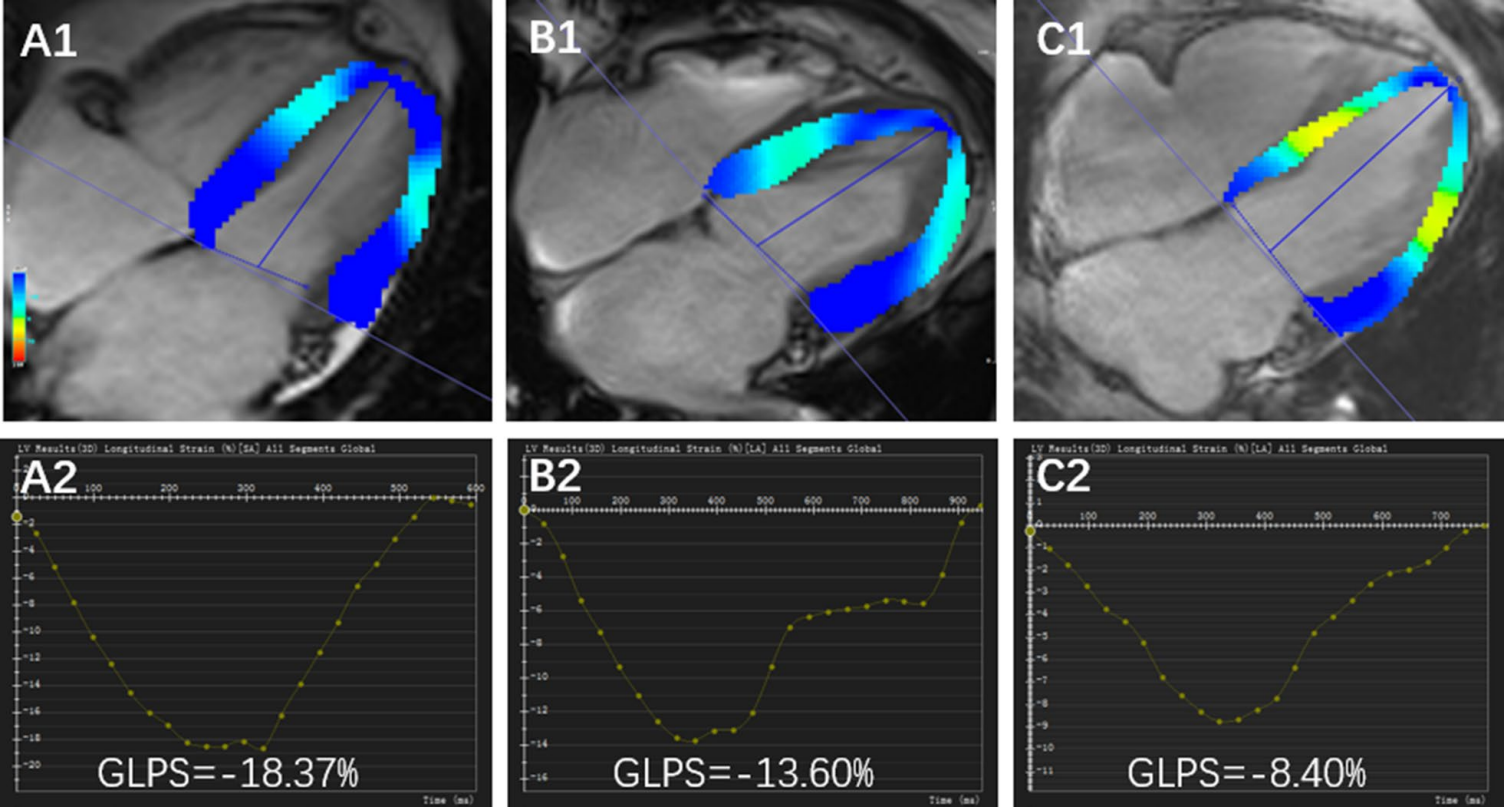
Representative CMR imaging LV pseudo color images of long-axis four-chamber cine images at the end-systole and CMR imaging derived global longitudinal peak strain curves. A1–2: a control subject, B1–2: a patient after acute MI without DM, C1–2: a patient after acute MI with DM

For LV myocardial deformation analysis, LV long-axis cine images (2-chamber and 4-chamber) and short-axis cine images were loaded into the feature tracking module. LV endocardial and epicardial borders were delineated at the end-diastolic phases of all cine images. The LV global LV global radial peak strain (GRPS), global circumferential peak strain (GCPS), and global longitudinal peak strain (GLPS) were acquired automatically (Fig. 2). For LGE analysis, the hyper-enhanced myocardium area was defined as the MI area on the LGE short-axis images when the signal intensity was five standard deviations above the mean intensity of the normal myocardium [18]. We assessed the extent of the LGE regions involving the LV wall by dividing it into the interventricular septum, anterior wall, inferior wall, and lateral wall using the 16-segment model. Two radiologists evaluated the images separately, and if the results were inconsistent, they discussed and agreed on the result.

### Reproducibility analysis

To determine intra-observer variability, LV global myocardial strain and LGE parameters in 90 random subjects (including 65 MI patients and 25 control subjects) were measured twice within 1 month by one observer (Y, G). A second observer (R, S), who was blinded to the results of the first observer and clinical data, reperformed the measurements to assess the interobserver variability.

### Statistical analysis

Statistical analyses were performed with SPSS (version 23.0; IBM SPSS, Inc., Chicago, IL, USA) and GraphPad Prism (version 8.0, GraphPad Software Inc., San Diego, CA, USA). Data were expressed as the mean ± standard deviation (SD) or median interquartile range (IQR) for continuous variables and frequencies for categorical variables. Categorical variables are presented as numbers (percentages) and were compared using Fisher’s exact test or the chi-square test, as appropriate. Parameters among MI(DM−), MI(DM+) and control groups were compared by one-way analysis of variance (one-way ANOVA) followed by Bonferroni’s post hoc test (normally distributed variables) or the Kruskal–Wallis rank test (nonparametric variables), as appropriate. Spearman’s and Pearson’s correlation analyses were conducted to identify the relationship between LV myocardial strain and clinical indices. Pearson’s correlation was used between continuous variables, and Spearman’s correlation was used to analyses the rank correlation. Moreover, variables with a *p* value of less than 0.1 in the univariable correlation analyses and an absence of collinearity were then included in a stepwise multivariable analysis to identify the independent determinants of LV global peak strain parameters. A *p* value of < 0.05 was considered statistically significant.

## Results

### Patient characteristics

Overall, 208 MI patients and 71 controls were included in this study. Of the 208 patients after acute MI, 95 patients were identified as having DM, and 113 patients were classified as non-DM patients. The main clinical baseline characteristics of the study cohort are summarized in Table 1. Age, sex, BMI, systolic and diastolic blood pressure, serum indexes and cardiovascular risk factors were not significantly different between the observed groups (all *p* > 0.05). The NYHA functional class in the MI(DM+) group was decreased compared with that in the MI(DM−) group (*p* < 0.05). The left anterior descending artery was the most common culprit vessel in both the MI(DM−) group and MI(DM+) group (46 [40.71%] vs. 41 [43.16%], *p* > 0.05). There was a higher number of diseased vessels in the MI(DM+) group than in the MI(DM−) group (*p* < 0.05). Additionally, the NT-proBNP value was significantly higher in the MI (DM+) group than in the MI (DM−) group (*p* < 0.05), and there was no difference in troponin value between the MI groups.

**Table 1:** Baseline characteristics of the study cohort

	Control (n = 71)	MI (DM−) (n = 113)	MI (DM+) (n = 95)
Baseline characteristics			
Age, years	58 ± 258.95	58.08 ± 1.88	60.76 ± 77
Male, n (%)	55 (77.46%)	97 (85.84%)	85 (89.47%)
BMI, kg/m^2^	23.31 (21.78, 25.27)	24.37 (22.31, 26.51)	25.16 (22.60, 27.22)
Systolic blood pressure, mmHg	127.83 ± 16.19	122.99 ± 19.31	126.90 ± 22.44
Diastolic blood pressure, mmHg	76.58 ± 10.16	75.17 ± 13.18	77.45 ± 11.83
Heart rate, bpm	70.69 (63.59, 78.97)	70.44 (61.79, 80.22)	73.19 (65.81, 80.97)
Cardiovascular risk factors			
Previous/current smoker, n (%)	–	77 (68.14%)	60 (63.16%)
Hyperlipidemia	–	65 (57.52%)	55 (57.89%)
Hypertension	–	50 (44.25%)	57 (60.00%)
Pervious PCI, n (%)	–	47 (41.59%)	43 (45.26%)
Pervious GABG n (%)		0	2 (2.11%)
NYHA functional class, n			
I	–	33 (29.20%)	12 (12.63%)^b^
II	–	41 (36.28%)	45 (47.37%)
III	–	33 (29.20%)	31 (32.63%)
IV	–	6 (5.31%)	7 (7.37%)
Culprit vessel n (%)			
Left main	–	3 (2.65%)	2 (2.11%)
Left anterior descending	–	46 (40.71%)	41 (43.16%)
Left circumflex	–	23 (20.35%)	18 (18.95%)
Right coronary artery	–	41 (36.28%)	34 (35.79%)
Number of diseased vessels n (%)			
1		58 (51.33%)	36 (37.89%)
2		34 (30.09%)	34 (35.79%)
3		17 (15.04%)	23 (24.21%)
HbA1c, %		5.75 ± 0.46	7.90 (7.00, 8.90)^b^
Triglycerides, mmol/L	1.43 (0.95, 1.83)	1.44 (1.04, 2.27)	1.43 (0.93, 2.01)
Total cholesterol, mmol/L	4.33 ± 0.54	3.55 (3.19, 3.91)	3.24 (2.89, 3.98)
HDL, mmol/L	1.32 ± 0.34	1.04 (0.84, 1.24)	1.00 (0.82, 1.16)
LDL, mmol/L	2.56 ± 0.58	1.26 (1.16, 1.96)	1.86 (1.51, 2.39)
eGFR, mL/min/1.73m^2^	100.32 (87.00, 110.85)	84.97 (72.37, 96.21)	80.31 (65.69, 94.55)
Troponin, ng/L		14.20 (9.40, 28.60)	20.90 (11.20, 39.10)
NT-proBNP	–	311.00 (167.50, 766.00)	687.00 (155.50, 1916.50)^b^
Concomitant medication, n (%)			
Aspirin, n (%)	–	106 (93.81%)	90 (94.74%)
ß-blockers, n (%)	–	92 (81.42%)	78 (82.11%)
ACEI/ARB, n (%)	–	68 (60.18%)	65 (68.42%)
Diuretics	–	46 (40.71%)	36 (37.89%)
Calcium-channel blocker	–	84 (74.34%)	74 (77.89%)
Insulin	–	12 (10.62%)	56 (58.95%)
Statin, n (%)	–	89 (78.76%)	89 (93.68%)

DM: diabetes mellitus; MI: myocardial infarction; BMI: body mass index; PCI: percutaneous transluminal coronary intervention; CABG: coronary artery bypass grafting; NYHA: New York Heart Association; HbA1c: glycated hemoglobin; HDL: high density lipoprotein; LDL: low density lipoprotein; eGFR estimated glomerular filtration rate; ACEI: angiotensin converting enzyme inhibitor; ARB: angiotensin receptor blockers

^a^: *p* < 0.05 versus control group (Bonferroni’s)

^b^: *p* < 0.05 versus MI patients without DM (Bonferroni’s)

### Comparison of LV function and global strain among MI patients with and without DM and controls

**Fig 3:**
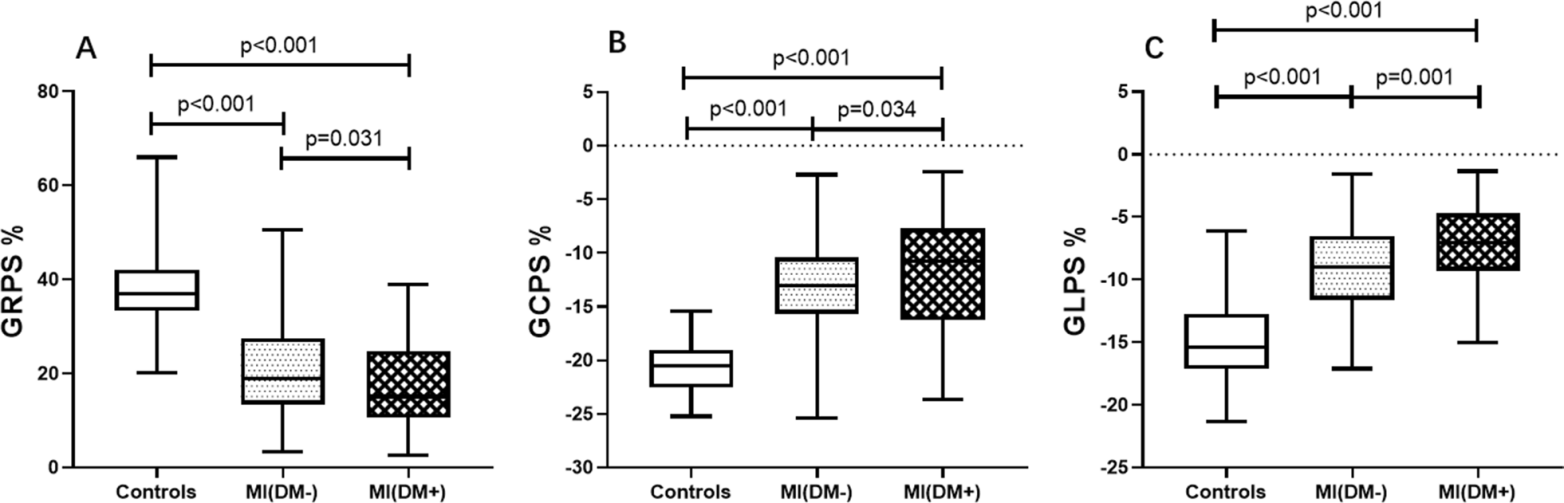
Comparison of LV global strains in three directions among MI (DM−) patients, MI (DM+) patients and control subjects

The CMR results for LV function and global peak strain are summarized in Table 2. In contrast to the control group, MI patients with and without DM exhibited an increased LVEDVi, LVESVi, LVMI, and decreased LVEF and LVGFI (all *p* < 0.05). The MI (DM+) group exhibited a higher LVMI and lower LVGFI than the MI (DM−) group (all *p* < 0.05), whereas the LVEF showed no difference between these two groups (*p* > 0.05). Regarding LV deformation parameters, all LV GRPS, GCPS and GLPS were decreased from in the controls to the MI (DM−) group to the MI (DM+) group (all *p* < 0.001, Fig. 3). In addition, the MI size of the LV was increased in the MI(DM+) group compared with the MI(DM−) group (24.38 (16.14, 33.46) % vs. 17.63 (10.94, 29.40) %, *p* < 0.05). There was no significant difference in MI territory in MI patients with or without DM (*p* > 0.05).

**Table 2:** CMR findings between controls, MI (DM−) group and MI (DM+) group

	Control (n = 71)	MI (DM−) (n = 113)	MI (DM+) (n = 95)
LVEDVi, mL/m^2^	72.89 (65.45, 83.05)	99.82 (78.57, 131.83)^a^	109.19 (86.83, 144.28)^a^
LVESVi, mL/m^2^	24.01 (19.78, 29.14)	54.78 (34.58, 84.41)^a^	64.52 (39.49, 98.89)^a^
LVSVi, mL/m^2^	49.08 (42.23, 53.77)	46.46 (38.77, 53.17)^a^	43.92 (35.41, 52.84)^a^
LVEF, %	65.40 (62.75, 70.01)	46.91 (34.34, 55.74)^a^	40.64 (28.88, 55.84)^a^
LVMI, g/m^2^	68.04 (60.20, 81.68)	101.06 (84.43, 126.98)^a^	114.23 (95.62, 134.13)^ab^
LVGFI	51.51 ± 6.89	34.33 ± 11.06^a^	30.71 ± 12.14^ab^
LV GPS, %			
Radial	36.95 (33.40, 41.89)	18.95 (13.69, 27.15)^a^	15.09 (10.52, 23.46)^ab^
Circumferential	− 20.55 (− 22.55, − 19.14)	− 13.05 (− 15.67, − 10.51)^a^	− 10.76 (− 16.15, − 7.78)^ab^
Longitudinal	− 15.40 (− 17.02, − 12.80)	− 9.04 (− 11.60, − 6.58)^a^	− 7.06 (− 9.21, − 4.76)^ab^
Infarct size, g % of LV	–	17.63 (10.94, 29.40)	24.38 (16.14, 33.46)^b^
Infarct territory, n (%)		–	
Interventricular septum	–	65 (57.52%)	53 (55.79%)
Inferior	–	43 (38.05%)	37 (38.95%)
Lateral	–	25 (22.12%)	29 (30.53%)
Anterior	–	32 (28.32%)	24 (6.12%)

Data are presented as median (25th, 75th percentile)

LVEDVi, left ventricular end diastolic volume index; LVESVi, left ventricular end systolic volume index; LVSVi, left ventricular stroke volume index; LVEF, left ventricular ejection fraction; LVGFI: left ventricular global function index; LVMI: left ventricle mass index; GPS: global peak strain

^a^: *p* < 0.05 versus control group (Bonferroni’s)

^b^: *p* < 0.05 versus MI(DM−) group (Bonferroni’s)

### Association of LV function and global strain with clinical variables in MI patients

Univariable and multivariable linear regression analyses were performed to evaluate the independent effect of DM on LV function and deformation in MI patients. After multivariable adjustment for covariates among all MI patients, DM was found to be an independent determinant of impaired LVGFI (β = 0.190, *p* = 0.004) and increased LVMI (β = 0.158, *p* = 0.021) (Table 3). Furthermore, systolic blood pressure, NT-proBNP level and infarct size were independently associated with LVGFI (β = 0.181, − 0.193, and 0.401, all *p* < 0.05). Age, hyperlipidemia and hypertension were independently associated with LVMI (β = − 0.230, 0.174 and 0.287, all *p* < 0.05) (Table 3).

**Table 3:** Determinants of LV dysfunction in MI patients

	LVGFI				LVMI			
	Univariable		Multivariable		Univariable		Multivariable	
	R	*p*	β	*p*	r	*p*	β	*p*
Age, years	− 0.029	0.674			0.197	0.004	− 0.230	0.001
Male, n (%)	0.105	0.131			0.147	0.034		
BMI, kg/m^2^	− 0.046	0.506			0.205	0.003		
Systolic blood pressure, mmHg	0.227	0.001	0.181	0.005	0.032	0.649		
Hyperlipidemia	0.073	0.294			0.178	0.012	0.174	0.012
Hypertension	0.124	0.143			0.210	0.002	0.287	< 0.001
DM	0.147	0.033	0.190	0.004	0.190	0.006	0.158	0.021
eGFR, mL/min/1.73m^2^	0.093	0.181			0.021	0.757		
NT-proBNP	− 0.230	0.001	− 0.193	0.003	0.152	0.030		
Infarct size, g % of LV	− 0.375	< 0.001	0.401	< 0.001	0.071	0.320		

Abbreviations as listed in Tables 1 and 2

NT-proBNP was log-transformed before being included in the regression analysis

After adjusting for confounding factors, the multivariable linear regression analysis showed that DM was independently associated with LV GRPS (β =  − 0.166, *p* = 0.007), GCPS (β = 0.164, *p* = 0.005) and GLPS (β = 0.262, *p* < 0.001) (Table 4). Moreover, NT-proBNP level, infarct size and LVMI were independently associated with LV GRPS (β =  − 0.140, − 0.375 and − 0.292, all *p* < 0.05), GCPS (β = 0.164, 0.431 and 0.316, all *p* < 0.05), and GLPS (β = 0.124, 0.300 and 0.331, all *p* < 0.05) (Table 4).

**Table 4:** Univariable and multivariable linear regression analysis of LV global peak strain in MI patients

	GRPS				GCPS				GLPS			
	Univariable		Multivariable		Univariable		Multivariable		Univariable		Multivariable	
	R	*p* value	β	*p* value	r	*p* value	β	*p* value	r	*p* value	β	*p* value
Age#, years	− 0.037	0.579			0.006	0.935			0.050	0.471		
Male, n (%)	− 0.138	0.047			0.153	0.027			0.091	0.190		
BMI, kg/m^2^	− 0.028	0.688			0.027	0.703			0.003	0.963		
Systolic blood pressure, mmHg	0.187	0.007	0.190	0.04	− 0.181	0.009	− 0.175	0.023	− 0.137	0.048		
DM	− 0.184	0.008	− 0.166	0.007	0.160	0.020	0.164	0.005	0.252	< 0.001	0.262	< 0.001
eGFR, mL/min/1.73m^2^	0.136	0.049			− 0.045	0.519			− 0.159	0.021		
NT-proBNP	− 0.198	0.004	− 0.140	0.021	0.185	0.008	0.164	0.004	0.194	0.005	0.124	0.041
Infarct size, g % of LV	− 0.387	< 0.001	− 0.375	< 0.001	0.459	< 0.001	0.431	< 0.001	0.308	< 0.003	0.300	< 0.001
LVMI, g/m^2^	− 0.262	< 0.001	− 0.292	< 0.001	0.273	< 0.001	0.316	< 0.001	0.260	< 0.001	0.331	< 0.001

Abbreviations as listed in Tables 1 and 2, NT-proBNP was log-transformed before being included in the regression analysis

### Comparison of LV global peak strain among MI (DM+) patients with good and poor glycemic control

**Fig. 4:**
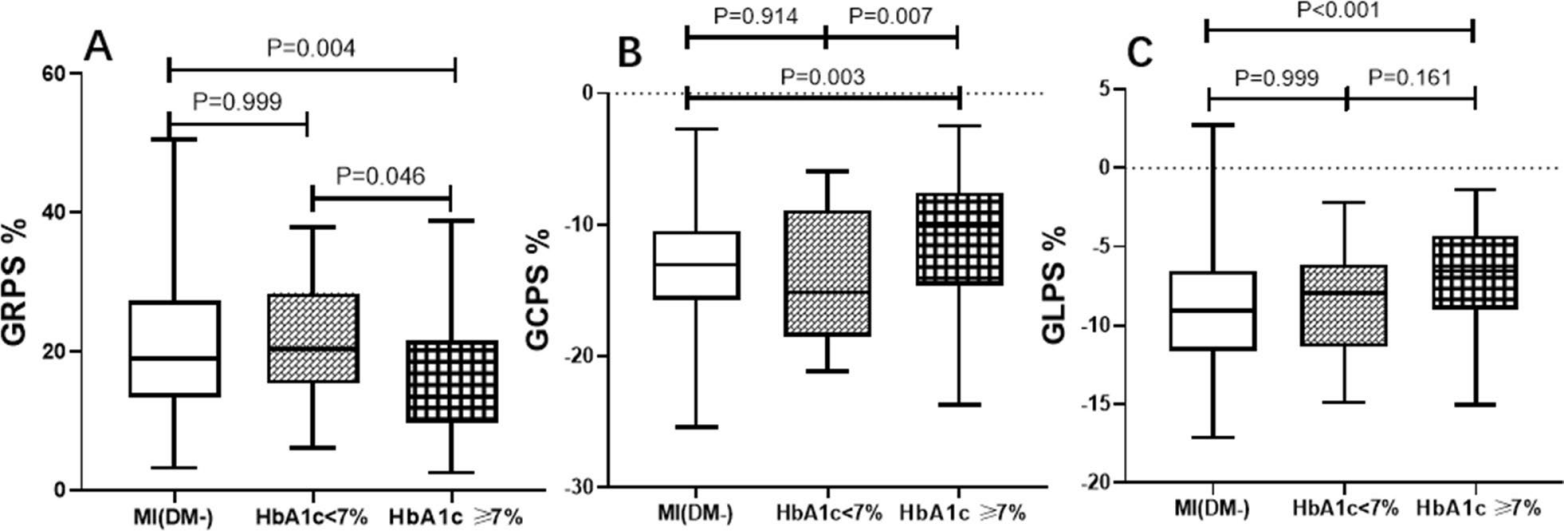
Comparison of LV global strains among MI (DM−) patients and MI (DM+) patients with good or poor glycemic control

According to the status of glycemic control, MI (DM+) patients were divided into two subgroups: good glycemic control (n = 23, HbA1c < 7.0%) and poor glycemic control (n = 72, HbA1c ≥ 7.0%). The LV global peak strain among MI (DM−) patients and MI (DM+) patients with good or poor glycemic control were shown in Fig. 4. MI (DM+) patients with poor glycemic control had a lower LV global peak strain in three directions than the MI (DM−) patients (all *p* < 0.001). There was no significant difference between MI(DM−) patients and patients with good glycemic control (HbA1c < 7.0%). LV GCPS was significantly decreased in poor glycemic control patients compared with good glycemic control patients [− 10.17 (− 14.56, − 7.60) % vs. − 15.13 (− 18.32, − 9.40) %, *p* = 0.014], whereas LV GRPS and GLPS showed a decreasing tendency. Moreover, there were increased LVESVi values and decreased LVSVi and LVGFI values in MI(DM+) patients with poor glycemic control compared with good glycemic control patients [LVESVi: 76.12 (43.05, 109.36) mL/m^2^ vs. 41.21 (30.91, 63.23); LVSVi: 43.48 (34.24, 49.32) mL/m^2^ vs. 49.13 (42.66, 57.98); LVGFI: 26.41 (18.83, 38.28) vs. 38.44 (30.63, 46.95); all *p* < 0.05].

### Independent effect of HbA1c on LV global peak strains in MI (DM+) patients

**Fig. 5:**
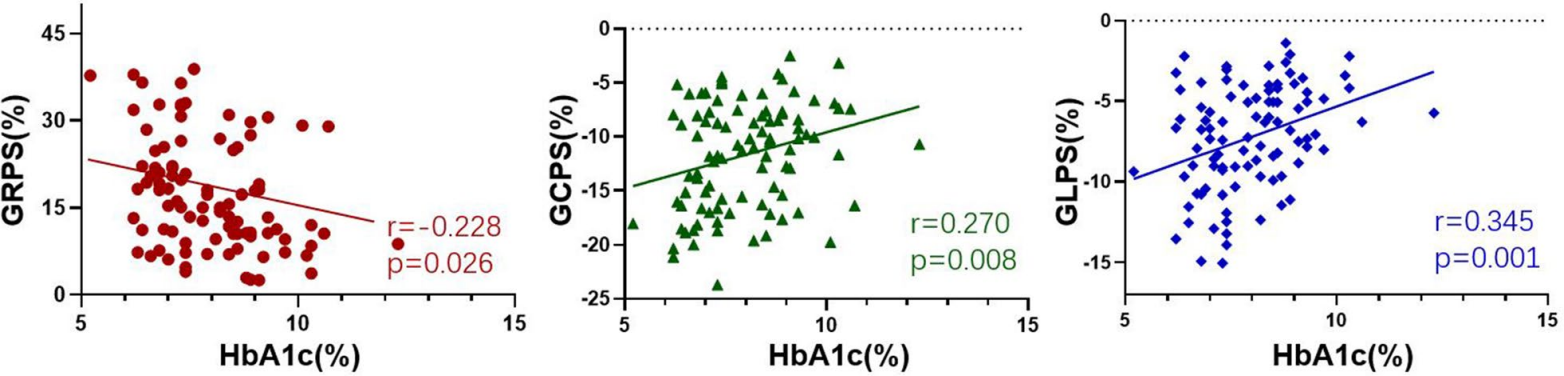
The associations between LV global peak strains and HbA1c level in MI (DM+) patients

The univariable analysis of MI (DM+) patients showed that HbA1c was negatively associated with GRPS (r =  − 0.228, *p* = 0.026) and positively associated with GCPS (r = 0.270, *p* = 0.008) and GLPS (r = 0.345, *p* = 0.001) (Fig. 5). After adjusting for confounding factors, HbA1c remained an independent determinant of impaired GRPS (β =  − 0.209, *p* = 0.025) and GLPS (β = 0.221, *p* = 0.010). Moreover, the log-transformed NT-proBNP level and infarct size were found to be independent determinants of global peak strain in all three directions (GRPS: β =  − 0.198 and − 387, GCPS: β = 0.290 and 0.552, GLPS: β = 0.227 and 0.308, all *p* < 0.01) (Table 5).

**Table 5:** Univariable and multivariable linear regression analysis of LV global peak strain in MI(DM+) patients

	GRPS				GCPS				GLPS			
	Univariable		Multivariable		Univariable		Multivariable		Univariable		Multivariable	
	R	*p* value	β	*p* value	r	*p* value	β	*p* value	r	*p* value	β	*p* value
Age, years	− 0.037	0.579			0.006	0.935			0.050	0.471		
Male, n (%)	− 0.138	0.047			0.153	0.027			0.091	0.190		
BMI, kg/m^2^	− 0.028	0.688			0.027	0.703			0.003	0.963		
Systolic blood pressure, mmHg	0.187	0.007			− 0.181	0.009			− 0.137	0.048		
HbA1c	− 0.228	0.026	− 0.209	0.025	0.270	0.008			0.345	0.001	0.221	0.010
eGFR, mL/min/1.73m^2^	0.136	0.049			− 0.045	0.519			− 0.159	0.021		
NT-proBNP	− 0.198	0.004	− 0.252	0.006	0.185	0.008	0.294	0.001	0.194	0.005	0.334	< 0.001
Infarct size, g % of LV	− 0.387	< 0.001	− 0.341	< 0.001	0.459	< 0.001	0.552	< 0.001	0.308	< 0.003	0.445	< 0.001

Abbreviations as listed in Tables 1 and 2

NT-proBNP was log-transformed before being included in the regression analysis

### Inter- and intra-observer variability

There was excellent intra- and interobserver agreement in terms of LV global strain and LV infarct size. The intra- and interobserver agreement was excellent for LV strain parameters (ICC = 0.923–0.978 and 0.912–0.961, respectively) and infract size of LV (ICC = 0.826–0.897 and 0.876–0.901, respectively).

## Discussion

This study investigated the combined effects of DM on LV function and deformation in patients after acute MI. The main findings of this study are as follows: (1) MI patients presented impaired LV function and deformation, whereas DM further deteriorated LV function and global peak strain in all three directions (radial, circumferential, and longitudinal); (2) For MI patients, DM was found to be an independent determinant of impaired LVGFI and LV global peak strain in all three directions; and (3) LV global peak strains declined progressively with the increase in HbA1c in MI patients with DM, and HbA1c was an independent determinant of decreased LV GRPS and GLPS. Our study indicated the deleterious effect of DM on LV deformation in patients with MI, and poor glycemic control may further aggravate the impairment.

DM, as the most common chronic metabolic disease, is the major risk factor for cardiovascular complications and adverse cardiovascular events. There is a high diagnosis rate of DM among patients with MI, and previous studies have reported a similar twofold increase in the risk for major adverse cardiovascular events in patients with DM after AMI [19, 20]. For patients after AMI, the absorption of myocardial edema and inflammation or fibrosis in the infarction core results in abnormal movement or adverse remodeling of the LV [10]. However, diastolic dysfunction and myocardial fibrosis have also been proven to be important damage stages in patients with DM. Although the conventional LV function parameters (i.e., LVEDVi, LVESVi, LVEF) were similar between the MI(DM−) and MI(DM+) groups, our study demonstrated that DM further impaired LVGFI and increased LVMI in MI patients. LVGFI is a CMR-validated measure of LV cardiac performance that integrates LV structure into LV functional assessment, which can provide incremental prognostic value for mortality after myocardial infarction. This finding reveals that the effects of DM on structural damage to the LV in patients after MI precede the decrease in LVEF [21, 22].

In this study, we conducted multivariable linear regression analysis and found that comorbid DM augmented the impairment of LV global peak strain in all three directions by CMR-FT in MI patients, and DM was an independent determinant of LV global peak strain in patients after MI. The underlying cause for cardiac alterations in patients after MI with DM is complex. Myocardial metabolism disorder is characteristic of patients with DM, and microvascular endothelial damage is aggravated in this microenvironment, leading to an increased incidence and severity of coronary atherosclerosis [23, 24]. In our study, patients with DM after MI had more coronary artery lesions, suggesting that myocardial ischemia may be more severe. Moreover, in patients with DM, the impairment of subendocardial fibers is aggravated, and these direct and indirect effects may partly explain the additive effect of DM on LV deformation in MI patients.

Blood glucose control is an important indicator to prevent adverse cardiovascular events in diabetic patients [25–27]. HbA1c, as an important biomarker of long-term blood glucose control in DM patients, is effectively and widely used to reflect the status of glycemic control. Previous studies have reported that the process of endothelial dysfunction and even myocardial fibrosis might be associated with hyperglycemia by accumulation of glycosylation end-products [28]. Admission glycemic variability has been identified as a predictor of mortality in patients with myocardial infarction, especially ST-segment elevation myocardial infarction [29]. Nystrom et al. reported that patients with type I MI with poor glycemic control (HbA1c > 7%) had a twofold higher risk of MACE than those with good glycemic control [29]. In the current study, our data showed that the decreased LV global peak strains were more prominent in patients after MI with DM. Multivariable linear regression analysis showed that HbA1c was an independent determinant of LV global radial and longitudinal peak strain in MI patients with DM. The infarction size and NT-proBNP level were strongly independently associated with LV GCPS, rather than HbA1c. We speculated that the influence of diabetes on myocardial compliance was mainly the longitudinal distribution of myocardial fibers in the subendocardial region. Since HbA1c is an independent determinant of LV global function and deformation, the status of glycemic control should be given more considerable attention in MI patients with DM.

Additionally, our study showed that NT-proBNP levels and infarct size were significantly higher in MI patients with DM than in those without DM, and these indices were independent determinants of LV global strains in MI patients with DM. Several clinical trials have demonstrated that NT-proBNP and infarct size are associated with reversed cardiac remodeling and dysfunction, which means that MI patients with DM have a more pronounced cardiac load and LV stiffness [30–32].

## Limitations

The study had several limitations. First, this was a retrospective single-center study, so there may be some selection bias in the results. Second, some MI patients underwent PCI and other operations, so the long-term effects of treatment on MI cannot be completely ruled out. However, there was no difference in the proportion of MI patients who received treatment between the two groups, and the possible deviation was reduced as much as possible. Third, we did not assess the type of MI in each patient, and future studies could be investigated with a larger cohort to evaluate the effects of different MI types on LV.

## Conclusions

In patients after MI, DM had an additive deleterious effect on LV myocardial strain. In addition, LV function and myocardial strain deteriorated with increasing HbA1c in these patients, which emphasizes the importance of glycemic control in MI patients.

## Data Availability

The datasets used and analyzed during the current study are available from the corresponding author on reasonable request.

## Abbreviations

MI: Myocardial infarction
DM: Diabetes mellitus
LV: Left ventricular
LVEF: Left ventricular ejection fraction
CMR: Cardiac Magnetic resonance imaging
LVEDVi: Left ventricular end diastolic volume index
LVESVi: Left ventricular end systolic volume index
LVSVi: Left ventricular stroke volume index
LVEF: Left ventricular ejection fraction
LVGFI: Left ventricular global function index
GRPS: Global radial peak strain
GCPS: Global circumferential peak strain
GLPS: Global longitudinal peak strain
BMI: Body mass index
HbA1c: Glycated hemoglobin
